# Impact of the COVID-19 pandemic on women’s contraceptive use: a mixed-methods study in South Africa and Zambia

**DOI:** 10.12688/gatesopenres.14590.1

**Published:** 2023-04-27

**Authors:** Rebecca L. Callahan, Alice F. Cartwright, Mags Beksinska, Margaret Kasaro, Jennifer H. Tang, Cecilia Milford, Christina Wong, Marissa Velarde, Virginia Maphumulo, Maria Fawzy, Manze Chinyama, Esther Chabu, Mayaba Mudenda, Jennifer Smit

**Affiliations:** 1 FHI 360, Durham, North Carolina, USA; 2 MatCH Research Unit (MRU), Department of Obstetrics and Gynecology, University of the Witwatersrand, Durban, South Africa; 3 Division of Global Women’s Health, Department of Obstetrics and Gynecology, The University of North Carolina at Chapel Hill, Chapel Hill, North Carolina, USA; 4 UNC Global Projects, Lusaka, Zambia

**Keywords:** family planning, contraception, COVID-19, South Africa, Zambia, Long-acting reversible contraception (LARC), LARC removal, mixed methods

## Abstract

**Background:** The COVID-19 pandemic affected global access to health services, including contraception. We sought to explore effects of the pandemic on family planning (FP) service provision and use in South Africa and Zambia, including on implant and intrauterine device (IUD) users’ desire and ability to obtain removal.

**Methods**: Between August 2020 and April 2021, we conducted surveys with 537 women participating in an ongoing longitudinal contraceptive continuation study. We also carried out in-depth interviews with 39 of the survey participants and 36 key informants involved in FP provision. We conducted descriptive analysis of survey responses and thematic analysis of interviews.

**Results**: Contraceptive use changed minimally in this sample with the emergence of COVID-19. Fewer than half of women (n=220) reported attempting to access FP since the start of the pandemic, the vast majority of whom were using short-acting methods. Among those who sought services, 95% obtained their preferred method. The proportion of women not using a method before and after pandemic start did not change in Zambia (31%); in South Africa, the proportion increased from 8% to 10%. Less than 7% of implant or IUD users in either country reported wanting removal. Among those who sought removal (n=22), 91% (n=10) in Zambia and 55% (n=6) in South Africa successfully obtained removal. In qualitative interviews, women with challenges accessing FP services mentioned long queues, deprioritization of contraceptive services, lack of transportation, stock-outs, and fear of contracting COVID-19 at a facility. Key informants reported stock-outs, especially of injectables, and staff shortages as barriers.

**Conclusions**: We did not find a substantial impact of COVID-19 on contraceptive access among this sample; however, providers and others involved in service provision identified risks to continuity of care. As the COVID-19 pandemic wanes, it continues to be important to monitor people’s ability to access their preferred contraceptive methods.

## Introduction

The emergence of the novel coronavirus, COVID-19, as a worldwide pandemic affected provision of and access to basic health services starting in early 2020 ^1, 2^. At the outset of the pandemic, public health and medical professionals urged governments and non-governmental associations to ensure continued access to family planning (FP) services ^3^. Strategies proposed in both developed and developing countries included expanding telemedicine for counseling and screening, sending prescriptions directly to pharmacies, increasing availability and supply of self-administered contraceptive methods (including through the outreach of community health workers (CHWs)), continuing to offer access to long-acting reversible contraception (LARC) as safety procedures allowed, and providing immediate postpartum and postabortion family planning care ^3– 5^.

Non-governmental organizations, governments, and donors also made calls to ensure that financing and logistic support for contraceptive commodities remained a focus to avoid stock-outs and ensure a range of methods were available in the event of supply chain disruptions ^5– 7^. In anticipation of possible limited access to removal services for long-acting reversible contraceptive (LARC) methods, such as contraceptive implants and intrauterine devices (IUDs), recommendations were also made that providers counsel users on method effectiveness beyond the labeled duration if they desired to continue their method ^3, 6^. The impact of COVID-19 was anticipated to have a particularly deleterious impact on access to FP services for people living in low and middle-income countries.

More than 36 months into the global pandemic, evidence has begun to emerge on the impact COVID-19 has had on access to contraception globally, particularly in the early days of the pandemic when national lockdowns and curfews were common. Data collected specifically among women in union from Kenya and Burkina Faso as part of the Performance Monitoring for Action (PMA) initiative found that the majority (67% in Burkina Faso and 82% in Kenya) did not change their contraceptive status between late 2019 /early 2020 and May–July 2020, though a small percentage did mention COVID-19-related reasons for not using any contraception ^8^. A population-level analysis of contraceptive need and use trends in Burkina Faso; Kenya; Lagos, Nigeria; and Kinshasa, Democratic Republic of the Congo using PMA data from before and after the start of the pandemic, determined that COVID-19 had not significantly impacted contraceptive need or use in these settings overall. However, some specific groups, such as nulliparous women, reported an increase in need, particularly in rural Burkina Faso and both rural and urban Kenya ^9^. Relevant to the study reported here, a prospective study of outpatient clinic visit patterns in KwaZulu-Natal, South Africa from January 27 ^th^ 2020 to April 29 ^th^ 2020 found reductions in child health visits after the national lockdown in March 2020, but no difference in FP visits ^10^. In contrast, another South African study in Gauteng Province documented a decline in the provision of injectable contraceptive methods and LARC, and an increase in oral contraceptive pills in the months leading up to and including the national lockdown compared to the year before ^11^. Concerns remained that sexual and reproductive health services could be impacted with additional waves of infection, and the manufacture of contraceptive commodities affected if pharmaceutical plants were forced to suspend production ^12^.

So far, few studies documenting providers’ experiences offering reproductive health services during the COVID-19 pandemic have been published. One exception was a survey of United States physicians, which found decreased LARC insertion and removal, but increased utilization of telehealth (the provision of health care services remotely using technology, such as a computer or mobile phone) for contraceptive counseling, renewal of prescriptions without an in-person visit, and allowing curbside pick-up or mailing of contraception ^13^. No changes in support of self-administration of subcutaneous injectable contraception, counseling on extended use of LARC, or advanced provision of emergency contraception or additional months’ supply of oral contraceptive pills were reported in this sample of providers.

The present analysis takes advantage of a unique sample of contraceptive users enrolled in a two-year longitudinal study of contraceptive use patterns to examine access to FP care during the COVID-19 pandemic in South Africa and Zambia. To explore more fully the factors surrounding supply, we also collected information from FP providers and others involved in service provision. The specific objectives of this study were to assess the effects of COVID-19 on family planning services and contraception access and use; to describe the effects of COVID-19 on LARC users’ desires and ability to remove their method; and to describe effects of COVID-19 on contraceptive supplies and service provision.

## Methods

### Ethics

All research participants gave written and verbal informed consent to participate in various aspects of the study. The main Contraceptive Use Beyond ECHO (CUBE) study, additional survey module, and in-depth interviews (IDIs) were approved by FHI 360’s Protection of Human Subjects Committee, the University of the Witwatersrand Human Research Ethics Committee (HREC), the University of North Carolina at Chapel Hill Institutional Review Board, and the University of Zambia Biomedical Research Ethics Committee (UNZABREC).

### Country contexts

In 2020 and 2021, COVID-19 was spreading globally, including across the African continent. South Africa had the highest number of documented COVID-19 cases in Africa at the time and roll-out of testing and vaccine availability, was slow the true impact may not yet be known in many countries. The first cases of COVID-19 were reported in South Africa on 5 ^th^ March 2020 ^14^ and in Zambia on 18 ^th^ March 2020 ^15^. The government of Zambia enacted a series of public health measures on 13 ^th^ March 2020, and the government of South Africa declared a National State of Disaster on 15 ^th^ March 2020, implementing stringent lockdown measures designed to control the spread of COVID-19. As of 10 ^th^ March 2023, South Africa had reported over 4 million cases and 102,595 deaths, while Zambia had reported 343,135 cases and 4,057 deaths ^16^.

### Study design

To better understand the experiences of accessing contraceptive methods within the context of COVID-19 lockdowns, we conducted a mixed methods study consisting of a survey module added to the CUBE study and IDIs with participants, FP providers, and other key informants involved with FP service provision. The methods for the CUBE have been described elsewhere ^17^. In brief, the CUBE study enrolled a subset of South African and Zambian women who had previously participated in the Evidence for Contraceptive Options and HIV Outcomes (ECHO) Trial, had agreed to be re-contacted, were using one of three contraceptive methods at ECHO trial exit, and consented to participate ^18^. These women were recruited to participate in a prospective longitudinal study (CUBE) after their exit from ECHO to explore contraceptive use dynamics and access to LARC removal services. The ECHO trial participants received either intramuscular depot medroxyprogesterone acetate (DMPA-IM), a levonorgestrel (LNG) implant, or a copper IUD for the 18-month ECHO trial period. Participants could switch or discontinue contraceptive methods both during the study period and at the time of study completion. For CUBE, participants were recruited from three of the 12 ECHO sites: two in the KwaZulu-Natal province in South Africa (an urban site situated in eThekwini District and a peri-urban site in uMgungundlovu District) and one in Lusaka, Zambia. Phone surveys were conducted with eligible women every six months for a total of four surveys over 24 months (at 6, 12, 18, and 24 months after ECHO exit). Since the COVID-19 survey module was initiated during the 24-month follow-up period, a portion of South African participants (less than 20%) were asked during their regularly scheduled 24-month call if they would be willing to participate in an additional module of questions related to COVID-19 during the current call or in a subsequent call. Most participants had already completed their 24-month survey and were re-contacted by phone and asked if they would be willing to complete the COVID-19 survey module. Surveys were conducted in South Africa between September 2020 and March 2021 and in Zambia from November 2020 to April 2021.

For the IDIs, interviews were conducted with a subset of CUBE participants along with FP providers and other key informants. Participant IDIs were conducted between September and November 2020 in South Africa and between April and June 2021 in Zambia. Key informant IDIs were conducted between August and October 2020 in South Africa and November 2020 and January 2021 in Zambia.

### Study population and sample

The main CUBE study enrolled 674 participants. For the COVID-19 survey module we sought to contact everyone who had completed the CUBE 18-month survey (n=626). Participants needed access to a phone to participate in both the CUBE study and the COVID-19 module. Written informed consent was obtained at initial CUBE enrollment and verbal consent was given to participate in the COVID-19 module. While the original eligibility criteria for the CUBE study was that participants had to be using one of the three ECHO contraceptive methods at enrollment (three-month injectable, LNG implant, or Copper IUD), there was no such restriction for participation in the COVID-19 survey module since participants could have switched from or discontinued the method they were using at CUBE enrollment. We were able to contact, consent, and interview 537 respondents for the COVID-19 survey.

A total of 39 CUBE participants (20 in South Africa and 19 in Zambia) and 36 key informants (16 in South Africa and 20 in Zambia) participated in the IDIs. IDI participants were purposefully sampled from the following four categories with an attempt to interview participants using all three contraceptive methods in each group (as applicable): 1) switched to a different method at ECHO exit; 2) discontinued their ECHO method while in CUBE; 3) continued their ECHO method into CUBE (and still using at 18 months); and 4) reported challenges getting LARC removal. For the key informant interviews, each country team created a list of FP providers, policy and program managers, and staff from FP commodity distribution centers in the public and NGO sectors, as well as other relevant stakeholders (e.g., NGOs, community advisory board (CAB) members, community advocates such as those representing sex workers, HIV-positive individuals, and youth groups). Key informant participants were purposively selected to represent local, district, provincial, and national levels.

### Data collection and analysis

***Phone surveys.*** Research assistants (RAs) called eligible women participating in the CUBE study by phone either for their regularly scheduled 24-month follow-up survey or, for those who had already completed their 24-month survey, an additional survey call. RAs described the additional COVID-19 module and obtained verbal informed consent to participate. Due to social distancing restrictions, informed consent for the additional module of COVID-19 questions was conducted over the phone. Study staff confirmed with the participant that they were free to talk and in an area that provided privacy, which was particularly important as partners may have been more likely to be home due to restrictions of movement related to the pandemic. If requested by the participant, information on COVID-19 and/or related services was provided, in line with South African Department of Health and Zambian Ministry of Health guidelines. At the end of the interviews, study staff in South Africa gave women the phone number for the South African COVID-19 hotline and staff in Zambia offered to send women a text message with all relevant contact information to get COVID-19 related updates.

The phone surveys were conducted in isiZulu or English in South Africa and Bemba, Chinyanja or English in Zambia and lasted between 5 to 114 minutes, with a median length of 20 minutes. Respondents were reimbursed in mobile money or phone credit which was sent to the mobile device of participants’ choice to offset expenditures related to use of their phones. RAs in each country entered participant responses into an online, password-protected RedCap database pre-programmed with response options to limit errors in data entry ^19^. Research staff in both countries and a research analyst at FHI 360, an international non-governmental organization, conducted weekly data quality checks. Any errors or inconsistencies were highlighted, and participants were recontacted as needed to correct responses.

***In-depth interviews.*** Trained female interviewers conducted the IDIs face-to-face, over the phone, or via a web-based platform such as Zoom depending on participant’s preference, distance from the study site, and social distancing/quarantine guidelines at the time of the interview. IDIs were conducted in isiZulu or English in South Africa and Bemba, Chinyanja, or English in Zambia and lasted approximately 20 and 45 minutes for CUBE participants and between 10 and 55 minutes for key informants. CUBE participants and key informants were reimbursed for their participation, with the exception of Department of Health staff in South Africa, in line with the Department of Health policy.

During the IDIs, CUBE participants were asked about issues associated with obtaining their contraceptive method of choice or any method; challenges in accessing FP services; method stock-out; gaps in use; method switching and discontinuation; and LARC removal. Key informant IDIs focused on contraceptive supply chain and procurement issues affecting method availability; effects of COVID-19 on provision of specific methods; and staffing and operational issues with FP service delivery including staff shortages, staff morale, safety concerns, and effects of social distancing. All IDIs were recorded and subsequently transcribed. If conducted in a language other than English, interviews were translated and transcribed simultaneously into English using a transcription protocol ^20^.

***Survey analysis.*** We linked each woman’s COVID-19 module to her full CUBE survey data (6, 12, 18 and 24-month survey responses), as well as additional demographic information reported in ECHO. We produced a descriptive summary of participant sociodemographic characteristics collected from ECHO, the 24-month CUBE survey, and the COVID-19 survey, as applicable. We compared sociodemographic characteristics between those who completed the COVID-19 module and those who were lost to follow-up. Using the dates of 24-month survey and COVID-19 module completion, we confirmed the contraceptive method that the participant was using before COVID-19 restrictions in their respective country. To assess the first two study objectives related to the effect of COVID-19 on contraception access and use and LARC users’ desires and ability to remove their method, we calculated descriptive statistics overall and by country. Summary statistics are provided, including percentages for categorical variables, and means/medians, standard deviations, and ranges for continuous variables.

***IDI analysis.*** We used applied thematic analysis to analyze data from the IDIs ^21^. A codebook was developed to structurally and thematically code the transcripts using NVivo 12 ^22^. Initially three analysts each coded the same transcript and then met to assess inter-coder reliability, discuss coding discrepancies, and to revise the codebook with emerging themes and to refine code definitions and inclusion/exclusion criteria. This process was repeated until inter-coder agreement was reached. Thereafter, analysts proceeded to code all the transcripts with frequent meetings held to ensure coding decisions were made jointly. After all transcripts were coded, coding reports were generated through NVivo 12, and four analysts worked on synthesizing data from each coding report by conducting inductive thematic analysis to identify major trends and thematic domains.

## Results

Characteristics of respondents are shown in Table 1. Mean age was slightly younger in South Africa (27.8 years) compared to Zambia (29.6 years). Women in South Africa had higher levels of education, were more likely to report being a student or employed, and more likely to report that they were not currently living with their partner compared with women in Zambia. Those lost-to-follow-up were significantly less likely to be students and more likely to be living with their partner compared to those who completed the COVID-19 module, though there were no significant differences in age, education, or parity. More women in South Africa (90.1%) were currently using contraception at the time of the COVID-19 survey compared with women in Zambia (68.7%). About a quarter of South African and just over two fifths of Zambian participants had ever had a COVID-19 test.

**Table 1.** Respondent characteristics.

	South Africa (N=342) n (%)	Zambia (N=195) n (%)	Total (N=537) n (%)
**Age at COVID-19 survey** (mean, SD) ^a^	27.8 (3.9)	29.6 (4.7)	28.4 (4.2)
19-24	88 (25.7)	48 (24.6)	136 (25.3)
25-30	195 (57.0)	73 (37.4)	268 (49.9)
31-38	59 (17.3)	74 (38.0)	133 (24.8)
**Level of education** ^b^			
No schooling	0 (0.0)	13 (6.7)	13 (2.4)
Primary school	0 (0.0)	73 (37.4)	73 (13.6)
Secondary school, not complete	108 (31.6)	77 (39.5)	185 (34.5)
Secondary school, complete	148 (43.3)	25 (12.8)	173 (32.2)
Attended post-secondary school	86 (25.2)	7 (3.6)	93 (17.3)
**Parity** (mean, SD) ^b^	1.2 (0.9)	2.6 (1.3)	1.7 (1.3)
**Employment status** ^c^*			
Housewife/Unemployed/Other	183 (53.8)	134 (69.4)	317 (59.5)
Student	61 (17.9)	4 (2.1)	65 (12.2)
Part or full-time employment	96 (28.2)	55 (28.5)	151 (28.3)
**Partner status** ^c^			
Living together (married/unmarried)	20 (5.9)	172 (88.7)	192 (36.0)
Not living together (married/unmarried)	303 (89.1)	21 (10.8)	324 (60.7)
No current partner ^†^	17 (5.0)	1 (0.5)	18 (3.4)
Currently using contraception ^d^	308 (90.1)	134 (68.7)	442 (82.3)
Received a COVID-19 test ^d^	82 (24.0)	86 (44.1)	168 (31.3)
Tested positive for COVID-19 ^d^	7 (8.5)	1 (1.2)	8 (4.8)

^a^Calculated based on age at ECHO enrollment and date of COVID-19 survey; ^b^Collected at ECHO enrollment; ^c^Collected at CUBE 24 month survey; ^d^ Collected in COVID-19 survey; *2 missing from South Africa and 2 missing from Zambia; **2 missing from South Africa and 1 missing from Zambia; †Includes widowed/separated/divorced

**Figure 1.**
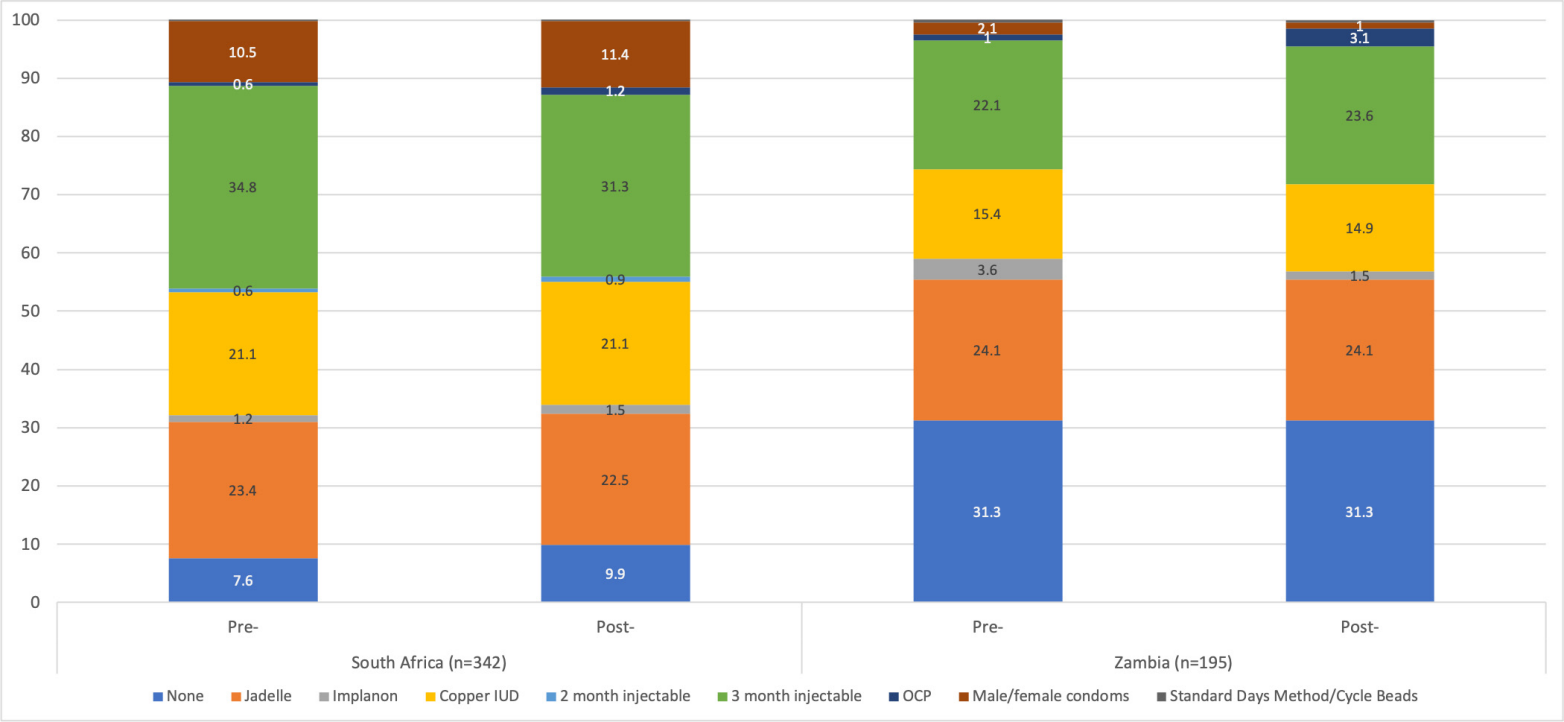
Methods used pre- and post-COVID-19 lockdown, by country (n=537).

Overall, 16% of respondents reported difficulty accessing sexual and reproductive health services (including FP and STI/HIV testing and treatment) since the beginning of the COVID-19 lockdowns and restrictions on movement ^23^. The proportion citing difficulties was higher in South Africa (23%) than Zambia (2%) (data not shown). Figure 1 shows the distribution of reported contraceptive method use before and after enactment of COVID-19 restrictions in each country. The proportion of women in South Africa who reported using no method increased marginally from the pre- to post-lockdown period. The proportion in Zambia was unchanged, but much larger overall (almost one-third of Zambian respondents reported not using any method in either period). In terms of method switching, slight reductions in IUD and three-month injectable use and increased use of condoms were reported in South Africa; and small decreases in IUD, implant, and condom use were reported in Zambia, along with slight increases in oral contraceptive pills (OCP) and three-month injectable use.

Half of COVID-19 module respondents across both countries had last obtained their method *before* COVID-19 restrictions went into place. However, the timing of method access was related to the type of method used. The vast majority of LARC users in both countries - 95% in South Africa and 93% in Zambia - obtained their method before COVID-19 restrictions, while all short-acting method users (with the exception of one condom user in Zambia) obtained their methods post-restrictions (not shown). For those who last obtained their method after COVID-19 restrictions came into place, 95% reported that they were able to get their preferred method, and only 4% (n=8) of those reported any impact of COVID-19 on their method use (not shown). For the 5% who were not able to obtain their preferred method post-COVID-19, it was almost exclusively because the method was out-of-stock.

Among all respondents, 14% reported switching or discontinuing their method after COVID-19 restrictions ( Table 2), and among those, only women in South Africa reported that COVID-19 restrictions affected why they switched or discontinued. All eight women in South Africa who reported that COVID-19 affected their contraceptive use were using three-month injectables prior to COVID-19. Seven of the eight reported the method was out of stock and one wanted to reduce her time in-person at the facility. Other reasons for changing methods included desire for a more reliable method (n=2) and concern that the facility would be closed due to COVID-19 (n=1).

**Table 2.** Switched or discontinued method after COVID-19 restrictions, by country.

	South Africa (n=342) n (%)	Zambia (n=195) n (%)	Total (n=537) n (%)
**Switched or discontinued method**			
No	291 (85.1)	171 (87.7)	462 (86.0)
Yes	51 (14.9)	24 (12.3)	75 (14.0)
**Did COVID-19 restrictions affect why switched method? (n=50)**			
No	28 (82.4)	16 (100)	44 (88.0)
Yes	6 (17.6)	0 (0.0)	6 (12.0)
Did COVID-19 restrictions affect why discontinued method? (n=25) *			
No	15 (88.2)	7 (100)	22 (88.0)
Yes	2 (11.8)	0 (0.0)	2 (8.0)

*1 missing from Zambia

Respondents were also asked about pregnancy and pregnancy planning. Just under 4% of all respondents (n=20) reported that they were currently pregnant. Among those, three quarters reported that they wanted to get pregnant when they did, two wanted to wait until later, and three did not want to become pregnant at all. Of these mistimed pregnancies (n=5), three respondents (who were all using injectable contraception pre-COVID-19) reported that COVID-19 had impacted their ability to delay or avoid pregnancy because the method they wanted was not available or they had a fear of being infected with COVID-19 while obtaining services. Among those *not* currently pregnant (n=517), 4% reported that they wanted to further delay or avoid pregnancy as a result of COVID-19, and 3% were not sure if their plans had changed (not shown).

**Figure 2.**
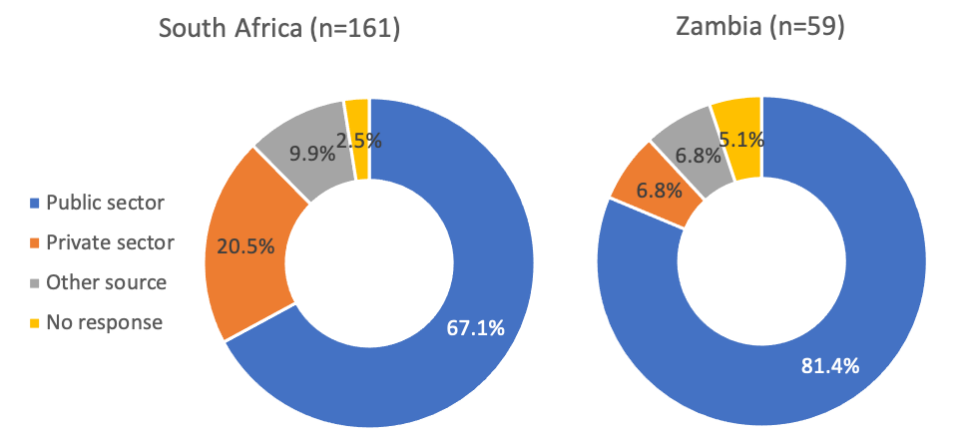
Location where methods were obtained post-COVID-19, by country (n=220).

Most respondents who were using a method and last obtained it after COVID-19 restrictions went into place, obtained their method from the public sector in both South Africa (67%) and Zambia (81%) ( Figure 2). These proportions were even higher (85% and 96%, respectively) when limited to injectable contraceptives (the most common method obtained after restrictions went into place). For those in South Africa who obtained condoms post-restrictions (n=39), 23% obtained them from the public sector, 37% from private health facilities, and 40% from another source (most commonly from a shop or market). Of the 220 women who last obtained their method post-COVID-19, 93% reported that COVID-19 did not play a role in where they obtained their method; rather people reported that they went to the location where they usually obtained FP and that this place was close to their home or work. For the 7% who reported COVID-19 related reasons (all in South Africa), the most common were hindrance of movement due to government restrictions or their usual source was not offering FP services (not shown).

For those respondents not using a method at the time of the survey (n=95), 14 said that they had wanted to obtain a method since COVID-19 and all but one attempted to obtain a method, mostly through the public sector. Most reported non-COVID-19-related reasons for not obtaining a method, including that they changed their mind (n=5), the method was not available (n=3), or they became pregnant (n=2). Only two respondents reported that they could not get a method due to FP services not being offered or a provider not available. Respondents who did not want to use a method were most commonly pregnant at the time of the survey or trying to get pregnant or not having sex, though a small number mentioned that they feared COVID-19 infection (n=2) or that they could not currently see their partner due to COVID-19 restrictions on movement (n=2). Finally, among all respondents, only 3% (n=14) reported wanting to change from their contraceptive method due to COVID-19, most commonly because they wanted a more reliable method (n=5) or wanted to reduce their time at the health facility (n=4). Smaller numbers were concerned about stockouts or facility closures due to COVID-19.

**Figure 3.**
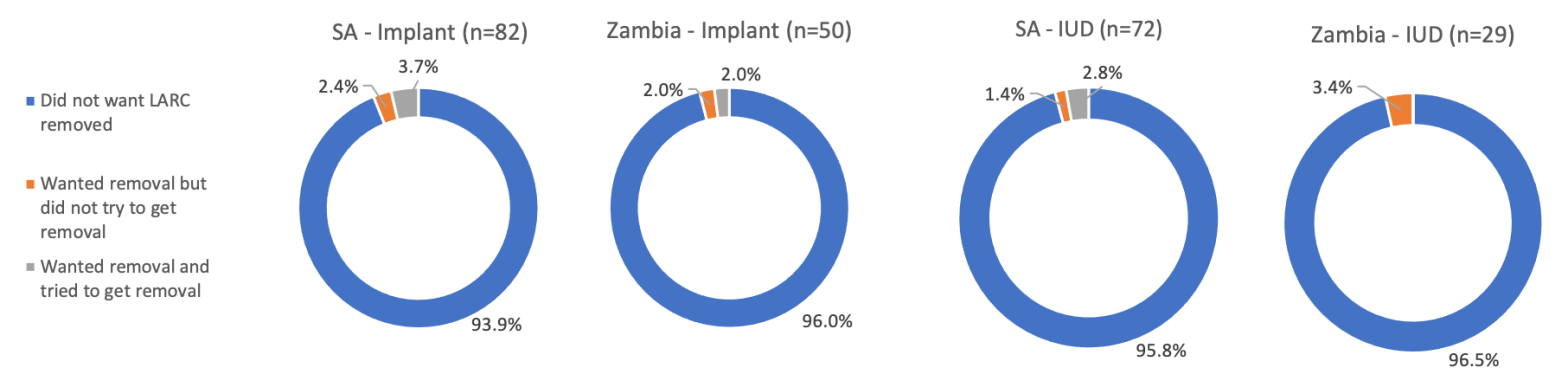
LARC removal desires and attempts, by country.

The overwhelming majority of LARC users at the time of the COVID-19 survey (n=233) had not wanted to get their method removed since restrictions went into place (>93% across both methods and country settings) ( Figure 3). Of the 11 LARC users who wanted to get their method removed post-COVID-19, six had attempted removal. Of these, three reported that they were unable to get removal because the provider told them to keep their method, one said a trained provider was not available, one reported that removal was too expensive, and one was told to go back to where the method was inserted for removal. Two of the six also said that COVID-19 had affected their ability to obtain removal. For those who wanted removal but did not try (n=5), reasons included the facility being closed or too full/busy, not being able to go to the provider due to COVID-19 restrictions, or changed their mind or partner wanted them to keep the method.

The proportion of successful LARC removals (respondents who wanted to have their method removed and obtained removal) was higher in Zambia (91%) than South Africa (55%) after COVID-19 restrictions went into place ( Table 3).

**Table 3.** Obtained LARC removal (among those who wanted and tried to get their method removed), by country.

	South Africa			Zambia			Total		
	Implant (n=8)	IUD (n=3)	Total (n=11)	Implant (n=8)	IUD (n=3)	Total (n=11)	Implant (n=16)	IUD (n=6)	Total (n=22)
Did not obtain removal	3 (37.5)	2 (66.7)	5 (45.5)	1 (12.5)	0 (0)	1 (9.1)	4 (25.0)	2 (33.3)	6 (27.3)
Obtained removal	5 (62.5)	1 (33.3)	6 (54.5)	7 (87.5)	3 (100)	10 (90.9)	12 (75.0)	4 (66.7)	16 (72.7)

### IDI results

***Impact of COVID-19 on access to reproductive health services.*** Of the 39 CUBE participants interviewed, 22 said they accessed reproductive healthcare services, including contraception and HIV/STI testing and treatment, after COVID-19 restrictions came into place. Of these, five South African participants said that they encountered challenges in accessing services, while no participant in Zambia reported challenges. As this South African participant explains, challenges included long queues at clinics and contraceptive services being deprioritized compared with other conditions:

Yes, it was not easy to go to the clinic since there is Corona. Because when you go to the clinic, firstly there will be long queues…They [ *healthcare workers*] will tell you to go home and wait till Corona is over. Because now they are dealing with people who are seriously ill…I just left it like that… ( **34 year old woman in South Africa, one previous live birth**)

Other challenges mentioned by South African participants (including those who did and did not attempt to access services) included limited capacity on public transport, the need to carry appointment cards or referral letters to travel during lockdown, and stock-outs of some contraceptives. 

Fear of contracting COVID-19 was also mentioned by participants and led at least three women in South Africa to avoid visiting a facility for services. In general, more South African participants reported fear of infection at a clinic compared to Zambian participants; however, the concern was raised in both countries. Fear of contracting COVID-19 led this South African woman to discontinue contraceptive use:

The COVID-19 has affected me in contraceptives because I have stopped using contraceptives because at the clinic it was full and I was scared that there will [ *be*] more chances to get infected with Corona if I go to the clinic the way it was full. ( **22 year old woman in South Africa, one previous live birth**)

Providers and other key informants in South Africa mentioned the same challenges that women described in accessing reproductive health services. In addition, providers and key informants also reported other challenges, including clients not able to get referral letters to other clinics because their usual clinic was closed, women desiring pregnancy termination but unable to access services, and method users finding it difficult to leave the house to get contraceptives during the lockdown because their partners did not know they were using contraceptives.

[ *Because*] most women were in lockdown with their partners you will find that it is hard to go out and get your contraceptive just because your partner does not know that you are taking contraceptive. Or he does not want you to take contraceptives, most especially we were getting those problems from those people who use pills most of the time. Someone will say, ‘it’s been five days and I am not able to take my pills because the partner is just there watching me all the time. I cannot hide because he is going to ask me, what is this pill for that you are taking?’ ( **Community advocate in South Africa**)

In both South Africa and Zambia, providers and key informants talked about the rise in unplanned pregnancies during lockdown. A key informant in Zambia shared how it has been particularly challenging for adolescents:

Even when there was no COVID, it was very difficult for young people to get sexual reproductive products. So when COVID came, it was double trouble because within the home they are not allowed. Culturally and traditionally they’ll not be allowed to ask questions about sexual issues, they don’t get information from their parents. So they don’t have, where during COVID, they did not interact with their peers, or to be able to hear some information from schools. ( **Community advocate in Zambia**)

***Impact of COVID-19 on family planning use.*** When COVID-19 restrictions were implemented in March 2020, 29 of the 39 CUBE participants who participated in IDIs were using a method, most commonly injectables, followed by implants, IUDs, condoms, and oral contraceptive pills. Those who were not using any contraceptive said that they were pregnant or that their partner was not staying with them during the lockdown. Six of the 29 method users (four in South Africa and two in Zambia) said they had switched or discontinued use after COVID-19 restrictions were implemented. Of the four South African participants, three said that they were not able to get their method because facilities were either closed or had no stock of their preferred method, or that they were afraid of going to the clinic and contracting COVID-19. The fourth switched methods due to menstrual side effects. The two participants from Zambia switched from their implants to other methods for personal reasons not influenced by the pandemic.

Of the 23 CUBE participants who had not switched methods since the start of the pandemic, about half (n=12) were using LARC. These users, especially in South Africa, said that they were glad they were on a long-acting method, so they did not need to visit a clinic. Several FP providers in South Africa also reported that women asked to switch to a LARC method during the pandemic, and others reported that women were asking for tubal ligations so that they would not have to go to a clinic, as this provider explains:

And also because they [ *clients*] would not be able to go out every month and buy the pill, especially under lockdown [ *level*] five and lockdown [ *level*] four, so I’ve seen an increase in long-term methods, I’ve seen women who are twenty, twenty-five [ *years old*], young women asking for permanent method of sterilization. ( **OB/GYN in South Africa**)

Overall, FP providers and other key informants described more negative impacts on FP use in South Africa compared to Zambia. Most Zambian key informants reported that services had largely not been interrupted and that women were not discontinuing use during the pandemic, some surmising that women did not want to become pregnant during the pandemic.

When CUBE participants were asked if COVID-19 had influenced whether they wanted to get pregnant in the next one to two years, seven out of 38 participants (two in South Africa and five in Zambia) said that COVID-19 had changed their plans about becoming pregnant. All seven said that the pandemic period was not the time to have a child because of the risk of the child getting infected with COVID-19, and that they would prefer to wait to become pregnant, as this Zambian participant explained:

Because things are not better right now, I would rather wait until things or like the pandemic goes. That’s when I can think of having children. ( **27 year old woman in Zambia, two previous live births**)

Several providers/key informants in South Africa and a few in Zambia reported that they feared an increase in unintended pregnancies, having already seen a rise in antenatal care since the start of the pandemic, as this Zambian provider shared:

Antenatal wise, we’ve seen also an increase in the first bookings of pregnancies. Meaning maybe people were scared to come to the facilities to get to the services for family planning. In the end, they ended up becoming pregnant because we’ve recorded like this time around, we are recording quite a high number of antenatal bookings. ( **Registered nurse in Zambia**)

***Impact of COVID-19 on contraceptive supplies and provision of family planning services.*** Providers and other key informants in both countries reported contraceptive stock-outs and other procurement issues as major challenges during the pandemic. Implants and injectables (both two-month and three-month) were the most commonly mentioned methods with procurement issues, but some also reported stock-outs for IUDs and pills. Some key informants said that procurement challenges were attributed to reduced transportation during lockdown, reduced patient flow because of restrictions, funding limitations, and competing priorities posed by the pandemic. In response to not having desired methods available, key informants noted that patients were often given another method, while less commonly noting that clients may be referred elsewhere or made to wait until their method was back in stock. A community advocate in South Africa explained how the pandemic affected method availability:

When you get there [ *FP clinic*] and you were told that the Depo[ *-Provera*] is not available, it is not available because the person that was supposed to order it forgot, she is more focused on COVID-19, and they will end up injecting you with the two months injection [ *norethisterone enanthate*]. ( **Community Advocate in South Africa**)

Many South African key informants also reported staff shortages, while only a few reported the same in Zambia. South African interviews described shortages due to infections among staff, time out for frequent testing, and the need to take on shifts in the COVID-19 wards. A few facilities addressed social distancing requirements by rotating staff on alternate days. Staff shortages led to the remaining staff feeling overwhelmed and increased client wait times. LARC services were sometimes not offered if trained staff were not present. Because of staff shortages in South Africa, one respondent said that they had to supplement services by bringing in Cuban doctors and referring patients to other facilities (usually in the private sector).

Providers and other key informants also reported that staff morale dropped because of the pandemic. In both countries many said staff were afraid of getting infected, with the fear causing further demoralization in their work. They also felt anxious when clients did not comply with safety guidelines, such as wearing masks:

It has affected staff morale, in that as we all know to say this is a deadly disease and having in mind that we have families where we are coming from, we don’t want to go back home and maybe come here get the infection, the virus, go back home, and go and give it to the family members. So, I’m sure it has affected the staff morale in some ways in that maybe some mothers, there are some clients that are coming in, are not complying to the regulations, like the guidelines, and also not knowing where those people are coming from. ( **Registered nurse in Zambia**)

Others felt that morale was low because providers were tired from a high influx of patients, staff shortages, having to self-isolate when infected, and practicing safe distancing at work which had been difficult as they could not shake hands, pray together, or give hugs to comfort patients/co-workers when they received bad news (e.g., the death of a family member).

***Impact of COVID-19 on LARC users’ desires and ability to remove their method.*** A few providers reported that demand for LARC removal decreased during the pandemic, with a few surmising that women were putting up with side effects longer (rather than seeking removal) because of the pandemic-related restrictions as this provider reported:

The services were available, but I think they just became a little bit more tolerant of their side effects, so they waited out of fear, until the lockdown came to Level 3, 2 and 1 before they presented with their side effects, so then many of the patients just tolerated it at home. ( **OB/GYN in South Africa**)

Others, however, said they had not seen a change in requests for either LARC removal or insertion. One provider in South Africa described greater challenges to LARC service provision in the public sector compared with the private sector due to pandemic lockdown restrictions.

## Discussion

In this sample of contraceptive users who had been followed prospectively over more than two years, we generally did not find a substantial impact of the COVID-19 pandemic on respondents’ ability to access their preferred contraceptive methods or LARC removal. Our findings are in line with findings to date on the minimal impact of COVID-19 on contraceptive use in South Africa and other sub-Saharan country settings ^9, 10^. However, interviews with providers and other key informants involved with FP service delivery revealed negative effects of the pandemic on provision and uptake of FP. Overall, we documented higher contraceptive use among our sample in South Africa compared to Zambia both before and after COVID-19 restrictions were implemented. The proportion not using a method during the pandemic rose slightly in South Africa compared to pre-pandemic levels but stayed flat in Zambia.

Encouragingly, we found that most survey respondents who *wanted* to use a method were currently using one. Among the small number (n=14) who were not using a method but wanted to, COVID-19 was not the primary reason reported for non-use. Rather, users cited changing their minds, becoming pregnant, or the method they wanted was not available. While providers and other key informants did describe negative effects of the pandemic on contraceptive supply chains, stock outs were identified as a problem pre-pandemic ^17^. In addition, we did not find extensive evidence of switching or discontinuation due to COVID-19 restrictions, again in line with what has been documented in other sub-Saharan African countries ^8^. To our knowledge, ours is the first study to inquire specifically about desires and attempts to obtain LARC removal in the COVID-19 period and we found that the overwhelming majority of LARC users in our sample did not want to get their method removed and most who sought removal were able to access services. 

While several FP providers and other stakeholders mentioned in the IDIs that demand for LARC methods had increased, we did not see an increase in LARC adoption in the survey data among women who were not using a LARC pre-pandemic. This could be related to the de-prioritizing of more intensive family planning services at health facilities during the early months of the pandemic, which was mentioned by providers and women alike. Almost all respondents who obtained a method after COVID-19 restrictions had gotten a short-acting method since they generally have to be obtained every 1-3 months. The majority seeking short-acting methods were still able to get them in the public sector. It may also be that due to their involvement in both the ECHO and the CUBE studies over the previous three and a half years, these women had had multiple opportunities to switch to a LARC method and were satisfied with their short-acting methods. However, it is possible that use patterns and method access continued to evolve as COVID-19 infections persisted. In the face of resurgence of the pandemic in the future, more people may want their LARC removed or face challenges in accessing their preferred short-acting methods if health facilities again become crowded or focused on managing COVID-19 patients.

### Limitations

One of the limitations of this study is that it constitutes a unique sample. Due to their participation in the ECHO and the CUBE studies, participants likely had more knowledge about contraceptive methods and where to obtain services. In addition, their use of LARC methods was much higher (40–45% of the sample across both countries) than the general population of women of reproductive age in either South Africa or Zambia. However, such a large number of LARC users allowed us to explore the impact of COVID-19 on method switching and discontinuation, specifically the ability to access LARC removal. Since only women were enrolled as participants in the original ECHO and CUBE study samples, we are unable to comment on how COVID-19 may have impacted men’s contraceptive use patterns. In addition, the participants were from ECHO study sites in urban and peri-urban areas. It is possible that the impact of the COVID-19 pandemic may have been more severe in rural areas.

The surveys and interviews described here were completed at slightly different points during the pandemic: between August 2020-March 2021 in South Africa and November 2020-June 2021 in Zambia. These time periods were after the initial wave of cases in Africa (particularly South Africa) and during the summer in the Southern hemisphere ^16^, when cases were likely lower overall. The experiences shared by respondents in this study may be subject to some recall bias and they may not reflect the situation as the pandemic progressed. Finally, the impact of the restrictions on family planning services in both countries was likely dynamic and possibly changed over time as the pandemic persisted.

## Conclusion

Among this group of contraceptive users in South Africa and Zambia, including a large number of LARC users, COVID-19 did not have a detrimental impact on contraceptive access, at least in the early days of the pandemic. However, interviews with family planning providers and other key stakeholders paint a slightly different, and more precarious, picture of the reproductive health care situation for the population at large. It will be important to continue to monitor the effects of the COVID-19 and other pandemics on health systems and access to primary services including family planning. Studies such as this one should be repeated to ensure that potential problems are identified proactively and access to contraception is protected.

## Data Availability

Full transcripts are not available for ethical reasons, to ensure anonymity of participants. However, relevant excerpts are available from the corresponding author. ECHO data is available for researchers who provide a methodologically sound proposal, which will be reviewed by the ECHO Management Committee. Proposals should be directed to icrc@uw.edu and data requestors will need to sign a data access agreement. Harvard Dataverse: CUBE Study COVID-19 Survey https://doi.org/10.7910/DVN/TJKRU4 ^24^ This project contains the following underlying data: CUBE COVID_Participant Survey.tab Harvard Dataverse: CUBE Study COVID-19 Survey https://doi.org/10.7910/DVN/TJKRU4 ^24^ This project contains the following extended data: CUBE COVID_Participant Survey_codebook.pdf CUBE COVID IDI Guide_Participants SA.pdf CUBE COVID IDI Guide_Participants ZM.pdf CUBE COVID IDI Guide_Key Informants SA.pdf CUBE COVID IDID Guide_Key Informants ZM.pdf Data are available under the terms of the Creative Commons Zero "No rights reserved" data waiver (CC0 1.0 Public domain dedication).

## Software availability

RedCap software was used for programming and recording participant responses to the COVID-19 survey. A similar program available at no cost is Open Data Kit. NVivo software was used for coding and thematic analyses of qualitative data. A similar program available at no cost is Taguette.
