# Supplementary material for: Contraceptive access and use before and during the COVID-19 pandemic: a mixed-methods study in South Africa and Zambia
Source: Gates Open Res. 2024 Jul 18;7:61. Originally published 2023 Apr 27. [Version 2] doi: 10.12688/gatesopenres.14590.2 (PMC11290906; doi:10.12688/gatesopenres.14590.2)
Supplement: Peer review history

# Reviewer report: Reviewer response for version 2

**DOI:** 10.21956/gatesopenres.17563.r37482

**Published:** 2024-07-27

**Authors:** Kamil Fuseini

**Affiliations:** 1 Population Council, Accra, Ghana

I have reviewed the responses to my comments and suggestions, and I am satisfied with the responses.

Is the work clearly and accurately presented and does it cite the current literature?

Yes

If applicable, is the statistical analysis and its interpretation appropriate?

Yes

Are all the source data underlying the results available to ensure full reproducibility?

Yes

Is the study design appropriate and is the work technically sound?

Partly

Are the conclusions drawn adequately supported by the results?

Partly

Are sufficient details of methods and analysis provided to allow replication by others?

Yes

Reviewer Expertise:

Population Health, Sexual and Reproductive Health (including family planning)

I confirm that I have read this submission and believe that I have an appropriate level of expertise to confirm that it is of an acceptable scientific standard.

# Reviewer report: Reviewer response for version 1

**DOI:** 10.21956/gatesopenres.15906.r35217

**Published:** 2023-11-14

**Authors:** Kamil Fuseini

**Affiliations:** 1 Population Council, Accra, Ghana

This paper titled “Impact of the COVID-19 pandemic on women’s contraceptive use: a mixed-methods study in South Africa and Zambia” sought to explore effects of the pandemic on family planning (FP) service provision and use in South Africa and Zambia, including on implant and intrauterine device (IUD) users’ desire and ability to obtain removal.

 The study is a mixed-method study conducted with a subset of participants from the ECHO study who were using either injectable Depo-Provera, LNG implant, or copper IUD at the end of the ECHO trial and who agreed to participate in a longitudinal follow up study (CUBE). The CUBE participants responded to an additional COVID-19 module, the dataset for this study. Key informant interviews were also conducted with selected key personnel in the FP space.

 The paper is clearly written, very informative and will make a good contribution to the literature; however, there are parts of the paper that can be strengthened.

1. The title of the paper is a little confusing as it suggests that the paper is looking at “… women’s contraceptive use …” yet looking at the objectives of the paper it seems to suggest that the paper is looking at various aspects of contraception including access. I suggest that the authors consider revising the title to include access.
2. The abstract needs to be edited. Parts of it do not read well. The conclusion of the abstract is also a little problematic as the title of the paper talks about use of contraceptives and the conclusion is talking about access. Which relates to my concern in comment 1.
3. The study design described in the paper is excellent, however, I do not think the statistical analytical methods are sufficient to assess “impact”. The analyses are descriptives and not sufficient to draw conclusions on the impact of an event. I would suggest that the paper be re-positioned and oriented. For example, change the title to “Use and access to contraceptive methods before - and during COVID-19 … ; Family Planning before and during".
4. The first paragraph of the study design does not also seem to suggest that the study is assessing impact and rightfully so, I do not think the analytical techniques are sufficient to do so. It would be clearer to revise the title to fit the content of the paper and make it clear that the paper is looking at use and access.
5. This section in the methods section “ … COVID-19 cases in Africa at the time and roll-out of testing and vaccine availability, was slow the true impact may not yet be known in many countries.” needs to be revised.

In general, the paper is a very good paper and if revised to reflect a descriptive paper, it would be great.

Is the work clearly and accurately presented and does it cite the current literature?

Yes

If applicable, is the statistical analysis and its interpretation appropriate?

Yes

Are all the source data underlying the results available to ensure full reproducibility?

Yes

Is the study design appropriate and is the work technically sound?

Partly

Are the conclusions drawn adequately supported by the results?

Partly

Are sufficient details of methods and analysis provided to allow replication by others?

Yes

Reviewer Expertise:

Population Health, Sexual and Reproductive Health (including family planning)

I confirm that I have read this submission and believe that I have an appropriate level of expertise to confirm that it is of an acceptable scientific standard, however I have significant reservations, as outlined above.

# Response

**Published:** 2024-07-08

**Authors:** Alice Cartwright

1. This is a good observation and suggestion.  We have changed the title of the paper to: **Contraceptive access and use before and during the COVID-19 pandemic: A mixed-methods study in South Africa and Zambia**

 2. We are not sure which parts of the abstract the reviewer refers to as not reading well, but we have made some edits, which hopefully are improvements.  We have adjusted the abstract conclusion and paper title to match.

 3. Thanks for this suggestion. We have changed the title of the paper as described above.

 4. We have addressed this through the title change.

 5. We have deleted this sentence.

# Reviewer report: Reviewer response for version 1

**DOI:** 10.21956/gatesopenres.15906.r35216

**Published:** 2023-11-03

**Authors:** Rebecca Geary

**Affiliations:** 1 University of Liverpool, Liverpool, UK

Thank you for the opportunity to review this paper on the "Impact of the COVID-19 pandemic on women’s contraceptive use: a mixed-methods study in South Africa and Zambia". The paper reports minimal impact of the COVID-19 pandemic on both contraceptive access, and LARC removal. 

 Among the survey sample of 537 women, only a small number reported difficulties accessing contraception, or wanting removal of their LARC method, limiting the sample and therefore inferences possible on the relative importance of different reasons for accessing contraception or removal. I would have welcomed the inclusion of confidence intervals to aid the interpretation of differences between countries, or pre- and post-COVID described in the text. I would also  recommend reviewing the presentation of small numbers in table cells to avoid presenting potentially disclosive results. These latter two points I consider important changes.

 Participants had previously participated in two other studies (CUBE and ECHO) and their survey responses for the current study were linked to those from CUBE and ECHO. It would have been helpful to have a figure illustrating which data used were from which study, and when and how those data were collected. 

 I was interested in the findings that varied between Zambia and South Africa and would have welcomed the author's reflections on potential reasons for these differences in the Discussion for those less familiar with these settings. I would also have welcomed more contextualisation of the results in the discussion in terms of the international literature.

Is the work clearly and accurately presented and does it cite the current literature?

Yes

If applicable, is the statistical analysis and its interpretation appropriate?

Partly

Are all the source data underlying the results available to ensure full reproducibility?

Yes

Is the study design appropriate and is the work technically sound?

Yes

Are the conclusions drawn adequately supported by the results?

Yes

Are sufficient details of methods and analysis provided to allow replication by others?

Yes

Reviewer Expertise:

Public health, contraceptive access, sexual health, health inequalities

I confirm that I have read this submission and believe that I have an appropriate level of expertise to confirm that it is of an acceptable scientific standard, however I have significant reservations, as outlined above.

# Response

**Published:** 2024-07-08

**Authors:** Alice Cartwright

1. In response to the reviewer’s comment, we have included confidence intervals around the proportions in Tables 1, 2, and 3. However, we would still like to remind the reviewer that this was a descriptive study, and while we present results from South Africa and Zambia to provide some degree of comparison, the study was not designed to draw conclusions about statistically significant differences.

 In response to the reviewer’s second comment, we have removed specific n’s from Tables 1, 2, and 3. In addition, for Figure 3 and Table 3, where the n’s and proportions are very small, we have combined those seeking implant and IUD removal into overall LARC removal to avoid any risks of deductive disclosure.

 2. For the sake of simplicity, we have not included a high level of detail about rates of participation in the ECHO and CUBE studies in this manuscript. We have acknowledged in the Notes to Table 1 the sources for the different pieces of information included. For timing of participation and other details on the methodology, we would refer the reviewer to these citations (included in the paper):

- ECHO study: Evidence for Contraceptive Options and HIV Outcomes (ECHO) Trial Consortium. HIV incidence among women using intramuscular depot medroxyprogesterone acetate, a copper intrauterine device, or a levonorgestrel implant for contraception: a randomised, multicentre, open-label trial. Lancet Lond Engl. 2019;394: 303–313. doi:10.1016/S0140-6736(19)31288-7
- CUBE study: Cartwright AF, Callahan RL, Beksinska M, Kasaro MP, Tang JH, Milford C, et al. Contraceptive Continuation and Experiences Obtaining Implant and IUD Removal Among Women Randomized to Use Injectable Contraception, Levonorgestrel Implant, and Copper IUD in South Africa and Zambia. Stud Fam Plann. 2023. doi:10.1111/sifp.12222

3. We have added mention in the Discussion section that the more stringent lockdown in South Africa seemed to have a more severe impact on accessing FP services including access to LARC removal: " While overall minimal, we did find a slightly greater effect of COVID restrictions on FP access and use, including access to LARC removal, in South Africa compared to Zambia.  This is likely because the lockdown measures in South Africa were more stringent than in Zambia, affecting access to and provision of methods."

# Reviewer report: Reviewer response for version 1

**DOI:** 10.21956/gatesopenres.15906.r34939

**Published:** 2023-09-28

**Authors:** Megan A Cohen

**Affiliations:** 1 Division of Complex Family Planning, Department of Gynecology and Obstetrics, Emory University, Atlanta, Georgia, USA

This is a mixed-methods study of the effect of the COVID-19 pandemic on women's access to, uptake, preferences for, and continuation of contraceptive use in South Africa and Zambia. It is very well written, timely, and of interest to an international audience. The study is performed on a subset of participants from the ECHO study who were using either injectable Depo-Provera, LNG implant, or copper IUD at the end of the ECHO trial and who agreed to participate in a longitudinal follow up study (CUBE). The CUBE participants were then also consented for the additional COVID-19 module which served as the dataset for this study. A subset of participants as well as key informants in the Family Planning sector were then also selected for key informant interviews. 

 The methods are adequately described and the study design appears to be able to answer the study objectives. The qualitative component and use of key informant interviews adds context to the study, particularly regarding the larger population who were not participants in the ECHO study, which the authors correctly identify as a special population with likely increased contraceptive knowledge compared to the population at large. 

 Overall the results are well-presented, but there were several areas of confusion or where I felt the presentation of the results and discussion could be strengthened. 

1. Do the 75 (14%) of individuals who stated they stopped or discontinued their contraceptive methods include the 20 pregnant patients (I assume so)? What is the breakdown by country of this, especially for the mistimed/unplanned pregnancies? Or are they separated? If they are not included in the individuals who stopped using a method, why not? They must have stopped given that they were pregnant?
2. Similarly, in the results on page 8 it states that 95 individuals were not using a method at the time of the survey, implying that more than 75 individuals either discontinued or switched methods. I think I may be missing that a certain proportion of individuals had already stopped using a method after ECHO but before the COVID-19 restrictions were in place? If this is the case, it would be very helpful to clarify this earlier in the results as the numbers do not seem to be adding up.  
3. Overall I would recommend moving down the numbers of participants into results, where that is traditionally reported. It may be helpful to have a Figure 1 or supplementary figure be how many out of the 626 who completed the CUBE 18-month survey were contacted, how many were not able to be reached, how many declined to consent to the COVID-19 module, how many were excluded if any for any reason, and then how many were ultimately enrolled. It could be helpful to also include at time of enrollment into the module if they were or were not using contraception and if so which type. It could be helpful to then also include how many were using contraception at the beginning of the enrollment for this module and how many were not. 
4. I'm confused about the coding of "method not available" or stock-outs as COVID-19 related in some places (i.e. in results on page 6 for South African women), but then not COVID-19 related later in the results (in the paragraph on the 95 patients not on contraception) and in the discussion. Is this specifically referring to a stock-out that occurred prior to COVID-19? It seems that if the method continued to be stocked out such that a patient could not reinitiate that would still be COVID-19 related, especially since it also came out as a theme in the Key Informant interviews. 
5. I agree that luckily most patients were able to continue their methods if desired and few sought to remove their LARC devices, but it does seem that there were some difficulties in South Africa that warrant a bit more discussion. Almost 50% of the individuals who desired LARC removal in South Africa were unable to obtain removal, which is a fairly big deal even though they were a small proportion of the study population. Additionally overall 23% of South African participants compared to 2% of participants in Zambia stating difficulties in accessing SRH services. It might be helpful to focus on this a bit more and perhaps place it into the context of what seemed to be more stringent lockdown in South Africa? Similarly it is a pretty big deal to have 3 out of 5 mistimed pregnancies as a result of COVID-related concerns or inability to obtain a method. So I might suggest a more nuanced discussion of the COVID-19 impacts in the discussion section, especially in regards to South Africa. 

Other thoughts:

1. The introduction is quite long. Could consider moving some of the results from other countries down into the discussion section to provide more context in the discussion and also to streamline the introduction. 
2. I would recommend that Figure 3 and Table 3 be organized in the same fashion. For example, consider presenting SA implant and SA IUD in the figure followed by Zambia implant and then Zambia IUD so it follows the same organization as the Table. 

Is the work clearly and accurately presented and does it cite the current literature?

Yes

If applicable, is the statistical analysis and its interpretation appropriate?

Yes

Are all the source data underlying the results available to ensure full reproducibility?

No source data required

Is the study design appropriate and is the work technically sound?

Yes

Are the conclusions drawn adequately supported by the results?

Partly

Are sufficient details of methods and analysis provided to allow replication by others?

Partly

Reviewer Expertise:

family planning, global contraception, obstetrics and gynecology

I confirm that I have read this submission and believe that I have an appropriate level of expertise to confirm that it is of an acceptable scientific standard, however I have significant reservations, as outlined above.

# Response

**Published:** 2024-07-08

**Authors:** Alice Cartwright

1. To clarify – the 75 individuals in Table 2 changed or discontinued their method after COVID-19 restrictions went into place (comparing the method they were using before COVID-19 restrictions and afterward). This was true for 10 of the 20 currently pregnant respondents. We take this to mean that the other 10 pregnant respondents discontinued their methods prior to COVID-19 restrictions but became pregnant after the restrictions went into place.

 Among the 10 pregnant respondents who stopped using their method after COVID-19 restrictions, only 1 South African respondent reported COVID-19 impacted their *method *use.

 However, when all of the 20 pregnant respondents were asked separately about the timing of their pregnancies post-COVID-19 restrictions, 15 of the 20 pregnant respondents (75%) reported that they wanted the pregnancy at that time, 2 said they wanted another pregnancy but wanted to wait until later, and 3 said they did not want to get pregnant at all. Among the 5 with mistimed or unwanted pregnancies (4 of whom were in South Africa), only 3 (all in South Africa) reported that they felt that COVID-19 restrictions impacted their ability to avoid or delay pregnancy (all 3 reported that the method they wanted was not available and 1 reported fear of being infected with COVID-19). We have described this group in the 6th paragraph of the Results section, but we have added some additional identifiers so that it is clear that the majority of mistimed or unwanted pregnancies were in South Africa.

 We acknowledge that it is challenging to align these slightly conflicting responses to different ways of asking the questions about pregnancy intention and contraceptive use, but ultimately 3 pregnant respondents, all in South Africa, reported that COVID-19 contributed to their mistimed or unwanted pregnancy. We have added a sentence addressing this to the Limitations section:

 “ Relatedly, with many factors affecting the health systems and contraceptive supplies, it is difficult to know if respondents accurately interpreted their situations as resulting from the COVID-19 pandemic. For example, in South Africa, stock-outs of contraceptive methods are not uncommon, and therefore, respondents may not necessarily see their inability to access their preferred contraceptive method as being related to COVID-19.”

 2. Table 1 captures the method that respondents are using at the time of the COVID-19 survey (where 95 respondents report they are not using a method). Before COVID-19 restrictions went into place, 70 respondents were already not using a method (and continued not using a method after restrictions went into place). We have added a sentence to clarify this in the second paragraph of the Results section: “ Most of the participants not using contraception had discontinued method use before COVID-19 restrictions went into place (n=70)”.

 Then from the time before to after COVID-19 restrictions went into place, 25 additional respondents discontinued their method (as reflected in Table 2). The 75 individuals in Table 2 either switched methods (n=50) or discontinued their method (n=25).

 3. We have moved the number of participants from the Methods to the Results section.

 We appreciate the suggestion to add an additional Figure; however, we are not sure how much additional information an additional Figure would provide to readers that could not be provided in the text. Therefore, we have added the following detail to the Methods in the "Study population and sample" section:

 “The main CUBE study enrolled 674 participants. For the COVID-19 survey module we sought to contact everyone who had completed the CUBE 18-month survey (n=626; n=390 in South Africa and n=236 in Zambia).”

 We have also added the following text to the beginning of the first paragraph of the Results section:

 “For the COVID-19 survey module, we were able to contact, consent, and survey 537 CUBE respondents. In South Africa, we were able to reach 87.9% of the 18-month CUBE participants, though one survey was only partially completed and excluded, resulting in a final sample of 342 respondents. In Zambia, we re-contacted 82.6% of the 18-month participants, resulting in a final sample of 195.”

 All participants were enrolled into the COVID-19 module *after* country-level restrictions had gone into place. As noted in the paper, the COVID-19 survey module was conducted between September 2020-April 2021. All information about their contraceptive use at the beginning of the COVID-19 module is provided in Figure 1.

 4. These points refer to slightly different groups. The former is those that switched or discontinued their methods *after* the date COVID-19 restrictions went into place and asks if respondents perceived that COVID-19 restrictions themselves impacted why they switched their method or discontinued. (5th paragraph in Results section and Table 2).

 The group of 95 people discussed in the 8th paragraph of the Results section are all of those who reported not using contraception at the time of the COVID-19 survey module. This includes the 25 who discontinued a method, but also includes those who were not using a method before the restrictions and continued not using a method. The results described in this paragraph are specifically focused on those who were not using a method but wanted to use one (n=14) to determine whether COVID-19 impacted their ability to *re-start* using a method. We found that for the majority of those people, COVID-19 was not the main determining factor in access.

 5. We have added mention in the Discussion that the more stringent lockdown in South Africa seemed to have a more severe impact on accessing FP services including access to LARC removal: " While overall minimal, we did find a slightly greater effect of COVID restrictions on FP access and use, including access to LARC removal, in South Africa compared to Zambia.  This is likely because the lockdown measures in South Africa were more stringent than in Zambia, affecting access to and provision of methods."

Other thoughts:

 1. We have moved references to other studies from the Introduction to the Discussion.

 2. We have combined the implant and IUD removal interest into LARC overall to align with Reviewer 2's concern about small n's and risk of disclosure. However, we have ordered Figure 3 and Table 3 such that South Africa and Zambia are presented consistently.
